# Characterisation of protein families in spider digestive fluids and their role in extra-oral digestion

**DOI:** 10.1186/s12864-017-3987-9

**Published:** 2017-08-10

**Authors:** André Walter, Jesper Bechsgaard, Carsten Scavenius, Thomas S. Dyrlund, Kristian W. Sanggaard, Jan J. Enghild, Trine Bilde

**Affiliations:** 1 Department of Bioscience, Aarhus University, Aarhus, Denmark; 2 Department of Molecular Biology and Genetics, Aarhus University, Aarhus, Denmark

**Keywords:** Digestive fluid, Spider, Venom, Proteomics, Astacin metalloproteases, Extra-oral digestion, *Stegodyphus*, *Acanthoscurria*

## Abstract

**Background:**

Spiders are predaceous arthropods that are capable of subduing and consuming relatively large prey items compared to their own body size. For this purpose, spiders have evolved potent venoms to immobilise prey and digestive fluids that break down nutrients inside the prey’s body by means of extra-oral digestion (EOD). Both secretions contain an array of active proteins, and an overlap of some components has been anecdotally reported, but not quantified. We systematically investigated the extent of such protein overlap. As venom injection and EOD succeed each other, we further infer functional explanations, and, by comparing two spider species belonging to different clades, assess its adaptive significance for spider EOD in general.

**Results:**

We describe the protein composition of the digestive fluids of the mygalomorph *Acanthoscurria geniculata* and the araneomorph *Stegodyphus mimosarum*, in comparison with previously published data on a third spider species. We found a number of similar hydrolases being highly abundant in all three species. Among them, members of the family of astacin-like metalloproteases were particularly abundant. While the importance of these proteases in spider venom and digestive fluid was previously noted, we now highlight their widespread use across different spider taxa. Finally, we found species specific differences in the protein overlap between venom and digestive fluid, with the difference being significantly greater in *S. mimosarum* compared to *A. geniculata*.

**Conclusions:**

The injection of venom precedes the injection with digestive fluid, and the overlap of proteins between venom and digestive fluid suggests an early involvement in EOD. Species specific differences in the overlap may reflect differences in ecology between our two study species. The protein composition of the digestive fluid of all the three species we compared is highly similar, suggesting that the cocktail of enzymes is highly conserved and adapted to spider EOD.

**Electronic supplementary material:**

The online version of this article (doi:10.1186/s12864-017-3987-9) contains supplementary material, which is available to authorized users.

## Background

Predaceous arthropods often face the ironic issue of being capable of subduing large prey without being able to mechanically break it up. Eighty percent of the known species tackle the problem by the use of extra-oral digestion, EOD [1], where the major break down of prey tissues takes place outside the predators’ body. The use of an array of digestive enzymes facilitates the consumption of the prey by liquefying its tissues before ingesting it. Spiders are one of the prominent examples for this curious adaptation. Moreover, they have almost exclusively evolved the use of silk to trap and immobilise prey. While free hunting species attack their prey directly and may wrap it in silk afterwards, web building spiders also use their silk to construct prey traps. The extraordinary mechanical strength of that material effectively absorbs the energy of struggling prey [2, 3]. Finally, many spiders are equipped with potent and biochemically complex venoms [4], which they use to sedate and kill prey items before consumption. The combination of EOD, silk and venom use allows these predators to exploit a wide spectrum of insect prey that can be many times larger than the attacking spider [5, 6].

Once captured and secured, the relative size of some insect prey and the rigidity of their exoskeleton limits the use of mechanical comminution to extract prey nutrients. Consequently, the evolution of EOD is tightly linked with the evolution of silk and venom use, and EOD in particular can be regarded as an expression of an extended phenotype [7]. Studies focussing on the behavioural adaptations to different life styles in spiders, cursorial or web building, diurnal or nocturnal, attack wrapping or bite attacking (based on venom use), are numerous. The molecular background with respect to the proteins used for silk, venom and digestive fluids, however, is still understudied. Which role do the different molecular components play in the realisation of the strategies used by the spiders? While proteomic techniques have already been deployed to progress our understanding of the composition, function and synthesis of spider silk [3, 8–11] and venom [4, 10, 12–14], investigations on the components and functionality of spider digestive fluids have only begun very recently [15]. Yet, several aspects of the feeding ecology of spiders prompt fascinating questions concerning the biomolecular composition of these fluids that may help us to understand the evolution of EOD. First, spiders can fast for a long period of time, which requires the active proteins in digestive fluids to be synthesised rather quickly or be reasonable stable over longer times. Second, the use of enzymes outside the protective milieu of the spiders’ body requires the incorporation of assisting proteins that regulate the activity and decelerate the degradation of active components in the open or in the unpredictable environment inside the prey’s body. Third, enzymes in digestive fluids need to be very potent as only very small amounts are produced and released by the spider to quickly dissolve a potentially large amount of prey tissues [16]. Finally, the mode of digesting extra-orally may have a beneficial side effect as it allows spiders to defend themselves against infections before potential pathogens enter their body. It may therefore not surprise if immune proteins are found in digestive fluids [15].

Insects are the main prey of spiders and are rich in proteins and lipids [17, 18]. Hence, spider digestive fluids are expected to be rich in proteases and lipases. In a pioneering study, the protein composition of digestive fluids of the orb web spider *Nephilingis* (*Nephilengys*) *cruentata* was investigated, and was shown to consist of a mix of hydrolases such as lipases and proteases, but also toxins and regulatory proteins [15]. A very abundant type of protein found was astacin-like metalloproteases (MEROPS peptidase database: http://merops.sanger.ac.uk/cgi-bin/famsum?family=M12) with 26 different copies present. Interestingly, this group of enzymes have also been found in spider venoms [19], suggesting that the venom may also have a digestive function [20]. As the venom injection usually precedes the release of digestive fluids it seems plausible that some of the venom components may pave the way to facilitate the effectiveness of the digestive enzymes [21, 22]. For example, Sanggaard et al. [10] identified hyaluronidases in the venom of the mygalomorph spider *Acanthoscurria geniculata*. These enzymes break down the extracellular matrix inside the prey’s body [23] for the toxins to be spread more quickly. However, the same effect is also applicable for facilitating the spread and efficiency of digestive enzymes injected after the venom. So far, the nature and number of proteins overlapping in the two secretions, digestive fluid and venom, have not yet been specifically analysed.

**Fig. 1:**
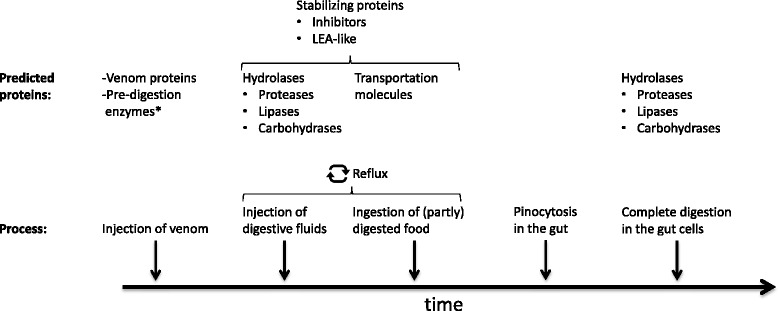
Scheme of the succession of steps of extra-oral digestion (EOD) in spiders with reference to the enzymes involved

The process of digestion in spiders is a complex succession of events that can be hypothesised to involve different kinds of enzymes at the different stages (Fig. 1). After physically attacking the prey the spider first injects venom into the prey’s body, a fluid that not only contains neurotoxic substances but also potential digestive enzymes [10, 20, 24]. Hence, the digestion process may be considered to start already at this stage before the release of digestive fluids. Depending on the size ratio of chelicerae and prey spiders either crush, or at least create punctures in the exoskeleton of the prey for the digestive fluids to enter. Subsequently, the release of a cocktail of digestive enzymes follows to dissolve the tissues of the prey (outside the spider’s body). As ‘refluxers’ [24], spiders then pump and suck digestive fluids and liquefied tissues back and forth between the prey and their gut. In that way, the carcass of the prey acts as a gut-like cavity where the digestive enzymes of the spiders can liquefy the tissues. In this part of the extra-oral digestion a variety of hydrolases starts to break down the prey tissues, including proteases, lipases, nucleases, carbohydrases, and the pumping reflux action improves the mixing and distribution of those enzymes [24]. However, as a prerequisite, special enzymes first need to break down the extracellular matrix that protects the prey tissue cells from being attacked by the hydrolases. Those ‘specialist proteins’ may be hyaluronidases [21], elastases, collagenases [24] or cysteine cathepsins [25, 26]. For the active proteases in digestive fluids, it may further be expected that they belong to the endo- rather than the exopeptidase family. Endopeptidases break the peptide bonds of nonterminal amino acids and therefore chop the prey proteins in smaller pieces, for them to be transported into the spider gut, where exopeptidases take over to more systematically disassemble the prey peptides by breaking the bonds of terminal amino acids step by step. The last part of the digestion can then be considered to be the pinocytosis of the various nutrients by the spider gut cells, followed by further intracellular break down [15].

**Fig. 2:**
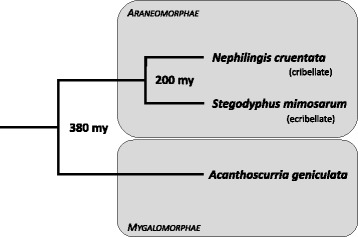
Phylogenetic position of our study species *Stegodyphus mimosarum* and *Acanthoscurria geniculata* in comparison to a third species used in a methodologically similar study by Fuzita et al. [15], *Nephilingis cruentata*. The latest common ancestor of both of our study species lived around 380 million years ago, demonstrating the long-time of independent evolution

Here we present proteomic analyses of the protein composition of the digestive fluid of two spider species representing different lineages within the spider phylogeny, the mygalomorph species *Acanthoscurria geniculata* and the araneomorph species *Stegodyphus mimosarum* (Fig. 2; see also phylogeny in [27]). If EOD has a common origin in spiders we would expect to find a similar protein composition in digestive fluids of both species, and similar to *Nephilingis cruentata* [15]. However, different prey capture strategies and dietary composition among spider species raises the question of whether adaptation to different dietary niches may lead to fine-tuned differences in protein composition of digestive fluids (see *Acanthoscurria*: [28]; *Stegodyphus*: [29, 30]). Our study species *A. geniculata* hunts without the use of silk, catching and subduing prey only by the use of their strong chelicera and the rapid injection of venom, while *S. mimosarum* represents a more derived species that uses composite silk threads (cribellate silk) to construct a capture web [31]. Moreover, the latter is also a social species where individuals build communal webs, engage in communal feeding and therefore shared EOD [32, 33]. By contrast, the *Nephilingis* species studied by Fuzita et al. [15] represents a highly derived, solitary orb weaving spider [31]. In contrast to the study of digestive fluids by Fuzita et al. [15], the novel aspect of our work is a thorough comparison of the compositions of these secretions in species with different dietary niches, distributed across the phylogenetic tree, while thereby particularly focussing on proteins being present in both, digestive fluids and venom. Previous studies only anecdotally reported that some venom proteins are also traceable in digestive fluids [15], yet the extent of the overlap is widely unknown. We systematically explore this issue in our two species, quantify their overlap, and infer functional explanations.

We extracted digestive fluids from our study species and used shotgun proteomic analyses to identify the protein compositions, which we compare between the study species and the data obtained by Fuzita et al. [15]. We hypothesise to find substantial overlap in the detection of specific enzymes among species, as their functionality and importance is expected to be highly conserved within the Araneae. However, we assess potential differences in composition and relative abundance of certain proteins related to the spiders’ prey capture strategy and prey preference. We discuss our findings in the context of the progression of extra-oral digestion and the relative importance of certain enzymes in each stage.

## Methods

### Sampling

We sampled digestive fluids from 9 adult *Stegodyphus mimosarum* females from nests that have been collected from a population in Kruger National Park, South Africa and brought to the lab at Aarhus University. Nine juvenile *Acanthoscurria geniculata* spiders (all of the same developmental stage) that were purchased from a pet store were sampled the same way for comparison. *Acanthoscurria geniculata* spiders were housed in individual plastic containers, and they were fed a cricket and watered once a week. The social *S. mimosarum* females were kept in their colonies, which were fed a mix of house flies and small crickets once a week. For the sampling one adult female per colony was chosen and taken out for digestive fluid sampling. In both species the sampling was conducted 7 days after the last feeding.

The spiders were first sedated in a chamber flooded with CO_2_ for 2 min. Subsequently individuals were fixated with gauze netting and pins on a Styrofoam block with the ventral side facing up. The spiders were then subjected to mild electric shocks from a stimulator (15 V/0.6A, Bang & Olufsen Power Supply SN16) by placing the electrodes at unsclerotised joint membranes of the coxae. This treatment caused muscles in the spiders’ prosoma to contract pressing digestive fluids out of the mouth. Under a binocular the regurgitated fluids were collected through a hole in the netting using a capillary. During the whole procedure the chelicerae of the spiders remained retracted from the mouth and fixated with pins in order to avoid a mixing of digestive fluids and venom. The release of venom through the tips of the chelicerae occurred only occasionally though, and the spatial separation of mouth and tips of chelicerae allowed a clear distinction of both fluids and uncontaminated sampling. Immediately after collection, digestive fluid samples were shock frozen in liquid nitrogen and later transferred to a − 80 °C freezer until the preparation for mass spectrometry analysis. The spiders were returned to their housing containers to recover and all equipment washed in 96% Ethanol prior to subsequent samplings.

### Mass spectrometry analysis and protein identification

All oral secretions from individual spiders were analysed by ‘the shotgun proteomics method’, which is based on liquid chromatography tandem mass spectrometry (LC-MS/MS). Proteins in the samples were precipitated using ethanol or TCA-precipitation protocols. Subsequently, the proteins were reduced in 8 M urea, 0.2 M Tris-HCl, pH 8 containing 10 mM DTT. Subsequently, the free cysteine residues were alkylated using 30 mM iodoacetamide. The reduced and alkylated sample was diluted five times and in solution-digested with trypsin (1/50 *w*/w) for 16 h at 37 °C. The resulting peptides were desalted by micro-purification using Poros 50 R2 reverse phase column material or C18 Empore Disk (3 M) and dissolved in 5% formic acid [34, 35].

Liquid chromatography-tandem mass spectrometry (nLC-MS/MS)-analysis was performed using a nano flow HPLC system (Thermo Scientific, EASY-nLC II) connected directly to the mass spectrometer (AB Sciex TripleTOF 5600+) equipped with a NanoSpray III source (AB Sciex) and operated under Analyst TF 1.6.0 control. The samples were injected, trapped, and desalted isocratically on a precolumn (2 cm × 100 μm, ReproSil-Pur C18-AQ 3 μm resin, Dr. Maisch). The peptides were eluted and separated on a 15 cm analytical column (75 μm i.d.), pulled in-house (P2000 laser puller, Sutter Instrument), and packed with ReproSil-Pur C18-AQ 3 μm resin (Dr. Maisch). Peptides were eluted from the analytical column at a flow rate of 250 nL/min using a 50 min gradient from 5% to 35% of solution B (0.1% formic acid, 100% acetonitrile). An information-dependent acquisition method, which acquires up to 50 MS/MS spectra per cycle at a 2.8 s cycle time and with an exclusion window of 10s, was employed.

All raw MS files were processed using Mascot Distiller 2.5.0 (Matrix Science). Peak picking were done using the default settings from the ABSciex_5600.opt file except that the MS/MS Peak Picking “Same as MS Peak Picking” was deselected and “Fit method” was set to “Single Peak”. After peak picking all scans, the data were searched against an *Acanthoscurria geniculata* or a *Stegodyphus mimosarum* database [10] using Mascot search engine (Matrix Science). The search parameters allowed one missed trypsin cleavage site, carbamidomethyl (C) as a fixed modification, and oxidation of methionine as variable modification. The mass accuracy of the precursor and product ions were set to 20 ppm and 0.1 Da, respectively, and a significance threshold of 0.01 was used. The Average [MD] quantitation protocol was selected using minimum 2 peptides, significance threshold at 0.01, matched rho was 0.7, XIC threshold was 0.1 and isolated precursor threshold was set at 0.5. The mascot distiller analysis results in an average peptide (ion) intensity for each quantified protein. The average intensities were converted to a relative protein amount by normalization. The relative values were calculated as the average peptide intensity divided by the total intensities of all quantified proteins. All samples were analysed in triplicates with the average relative amount reported.

## Results and discussion

The proteome of the digestive fluid from *Stegodyphus mimosarum* and *Acanthoscurria geniculate* were identified and quantified using relative quantification. In total, 527 proteins were identified in *Stegodyphus mimosarum* and 305 proteins in *Acanthoscurria geniculata*, out of which 71 and 37 respectively were quantified based on ion intensities (see Additional files 1 and 2). The lower number of detected proteins in *A. geniculata* may well have a biological basis, but it may alternatively derive from the lower quality of the *Acanthoscurria* genome assembly and thus suffer from more identification gaps. Apart from the proteins that we discuss in the context of either venom or digestive activities, we also found a number of cellular proteins in low concentrations that most likely derive from gut cells, since the spiders were not physically damaged during the digestive fluid sampling.

The protein compositions of both species’ digestive fluids revealed some overall similarities, which we discuss to account for their function to break down the matrix and tissues of insects, the main prey of spiders. Some of the steps in the digestion process, however, can be accomplished by the use of analogous enzymes, potentially explaining some of the differences we also detected while comparing our two study species with each other and with the data on a third species, *Nephilingis cruentata*, provided by Fuzita et al. [15]. In the following we present and discuss our findings referring to distinct protein families and the according stage of digestion they are likely to play a major role in.

### Immune-related proteins in the digestive fluid

Extra-oral digestion means that spiders are able to break down the nutrients of captured prey outside their own body. Accordingly, they can both digest large amounts of tissues and separate indigestible parts, such as the exoskeleton of the prey, before they ingest it. This has the beneficial side effect that EOD also allows for an early immune defence, potentially allowing spiders to reduce the risk of infections by preventing pathogens of entering their body.

#### Chitinases

In total we found eight chitinases in the mygalomorph *A. geniculata* and three in the araneomorph *S. mimosarum* (Table 1); a protein group that was also found in *N. cruentata* digestive fluids [15]. Given that spiders are predators that feed on arthropod prey with sclerotized exoskeletons, a high number of chitinases may not surprise. However, their appearance in the digestive fluids is not necessarily solely related to their function to cut through the cuticle of prey insects, but may also have a role in immune response regulation [36]. Many spiders mechanically break a hole into the cuticle of an insect by the use of their chelicerae and subsequently inject venom and digestive fluids [37]. If the relative size of the prey is too large for being crushed and macerated completely, spiders need the intact carcass of the prey as a ‘hard bowl’ to contain the liquefied tissues prior to ingestion. The latter is the case for our *Stegodyphus* species, and enzymatically attacking the exoskeleton seems counterproductive. Therefore, these proteins may have combined functions. Chitinases are also very potent in attacking cell walls of bacteria and fungi [36]. Since EOD is process that takes hours of close contact with the prey, we suggest that the chitinases act as a means of an immune response, functioning as an early deployed defence against pathogens to reduce the risk of infection.

**Table 1:** Overview of the proteins detected in digestive fluids of *Stegodyphus mimosarum* and *Acanthoscurria geniculata* in comparison with those found in *Nephilingis cruentata* by *Fuzita et al. [15]

*Species*		*Stegodyphus mimosarum*		*Acanthoscurria geniculata*		Nephilegys cruentata*	
Protein family		quantified	low concentration	quantified	low concentration	quantified	low concentration
Lipases		1	14	1	1	6	2
Carbohydrases	Alpha-amylase	1	1	-	-	1	-
	Alpha-mannosidase	-	1	-	-	1	1
	Glucose dehydrogenase	1	-	-	-	1	-
	Beta-hexosaminidases	-	-	-	2	1	1
	Enolase	-	1	-	-	1	1
	Beta-galactosidase	-	-	-	-	1	1
	Maltase-glucoamylase	-	1	-	1	-	-
	Glycolate oxidase	-	1	1	-	-	-
Proteases							
*Endo-*	Trypsin-like proteins	9	3	3	3	8	-
	Cysteine proteases	1	2	1	1	-	-
	Astacin-like metalloproteases	9	24	1	11	25	4
	Zinc-metalloproteases	-	1	-	-	1	-
	Cathepsins	1	4	-	3	3	-
*Exo-*	Carboxypeptidases	1	-	1	5	4	1
Nucleases		1	3	-	1	1	-
Transport and chaperon-like proteins	Lipid transport proteins	2	-	-	-	1	-
	LEA-like proteins	3	-	-	-	-	-
Immune response	Chitinases	2	1	5	3	3	1
	Peptidoglycan recognition proteins	1	-	-	3	2	-
	Cell adhesion molecule	-	1	-	-	1	1
	Lysozyme	-	1	-	1	-	-
Other proteins	Peroxidase	1	-	-	-	5	-
	Serine protease inhibitors (Serpins)	-	-	2	1	1	-
	Serine/threonine-protein phosphatase	-	1	-	-	1	1
	Carbonic anhydrases	-	1	-	-	2	-
	Alcohol dehydrogenases	-	-	1	1	2	-
	Pantetheinase	-	1	-	1	1	-
	Superoxid dismutases	-	1	-	-	3	1
	Leucine rich repeat proteins	7	7	-	4	6	1
	Allergens	5	-	2	-	4	-
Unknown proteins		20	*questionable*	18	*questionable*	76	*questionable*

Left column: quantified proteins; Right column: detected, but unquantifiable proteins. Of all proteins detected in *Nephilingis* we only included those that find comparable equivalents in our results. For a more detailed list see Fuzita et al. [15]

#### Other immune-related proteins

Apart from Chitinases we further detected three other groups of proteins potentially serving the immune response, a cell adhesion molecule (one in *S. mimosarum*), lysozyme (one in *S. mimosarum* and one in *A. geniculata*) and several peptidoglycan recognition proteins (two in *S. mimosarum* and three in *A. geniculata*). These proteins are crucial immune receptors, recognising viruses and bacteria prior to attacking them [38–41].

### Enzymes breaking down the extracellular matrix

One of the major difficulties with EOD in refluxers like spiders is that the prey tissues are not always mechanically shredded but need to be dissolved by the action of enzymes only [24]. Thus, once the spiders managed to transfer their digestive fluids into the prey’s body, enzymes are required to break down a second, internal barrier, the extracellular matrix, which mainly consists of fibrous proteins like collagen and hyaluronan, high molecular mass polysaccharides [42]. Accordingly, we expected to find hydrolases that disrupt this matrix and enable other digestive enzymes to reach the tissue cells. Hyaluronidases, however, could not be detected in digestive fluids of neither our study species nor *Nephilingis* [15]. This lack of this enzyme group may be understandable considering the fact that hyaluronic acid is not very abundant in insects, the main prey of spiders [43, 44]. However, in a study on *A. geniculata* a hyaluronidase was found in the venom of this species [10, 45], and its activity may benefit not only the spread of toxic components [46], perhaps especially in non-insect prey, but it may also facilitate the spread of digestive enzymes that are subsequently injected.

Apart from polysaccharides, there are fibrous proteins in the extracellular matrix that need to be broken down. Candidate enzymes were found in the form of various cathepsins (2× B, 1× D in *A. geniculata;* 3× L, 1× B, 1× D in *S. mimosarum*; cf. Tab. 1 and Additional file 1). In their study on *N. cruentata*, Fuzita et al. [15] also found cathepsins B and L, which were recently described to have an active function in dissolving the extracellular matrix [25]. Apart from the cathepsins, another enzyme that may help to break down the extracellular matrix was detected (but not quantified) in *S. mimosarum* only, elastase. Finally, some astacin-like metalloproteases, which we found in high abundance in our study species (see below), have also been described to process proteins of the extracellular matrix [47, 48]. Yet, the exact identification of those enzymes is difficult within the relatively large number of duplicates and without a functional annotation for each of them.

### Digestive enzymes

#### Carbohydrases

The main prey of spiders is insects, which are rich in protein and lipids [49], yet carbohydrases that break down polysaccharids are also found in the digestive fluids. For example, in our study an alpha-amylase was quantifiably found in *S. mimosarum*, while only traces of a similar enzyme were found in *A. geniculata* (Maltase-Glucoamylase). This enzyme has also been found in *Nephilingis* [15], and further similarities with digestive fluids of *S. mimosarum* could be seen in the presence of glucose dehydrogenases, Alpha-mannosidases and Enolases (Table 1). Mommsen [50] did an early biochemical characterisation of alpha-amylases in spiders and a more recent study by Eggs & Sanders [51] suggests that carbohydrases like those may help the spiders to digest pollen that is either caught in the web or attached to insect prey. That may explain the presence of these enzymes in the web building species, *N. cruentata* and *S. mimosarum*. In *Acanthoscurria*, however, only a few carbohydrases were detected, which may reflect the different nutritional content of its main diet, more strongly based on cursorial insects, like cockroaches, beetles and crickets [28, 29]. Yet the main fraction of carbohydrases in all three species consisted of the aforementioned chitinases.

#### Lipases

Next to proteins, lipids are major nutrients that predacious spiders exploit in insects [17]. In accordance, lipases were found to be numerous in their digestive fluids. In *S. mimosarum,* we detected 15 lipases with one quantifiable at a high percentage (2%) and the other in low concentrations (cf. Tab. 1). Interestingly, this abundance was not mirrored in *A. geniculata* where we only found two lipases. In *Nephilingis* [15] an intermediate number was detected, eight. The relative scarcity of lipases in digestive fluids of the mygalomorph species may be related to a more protein based prey spectrum [52]. The dietary preferences are species specific in spiders and can further vary with spider condition and feeding regime [17, 53]. Hence, the enzymatic composition of the digestive fluids may reflect adaptations to different diets. Similar differences can then also be found when looking at proteins that assist lipid digestion. In both *Stegodyphus* and *Nephilingis* lipid transport proteins are present, but these seem to be absent in *Acanthoscurria*. The detected apolipoproteins enclose lipids and create hydrophilic lipoproteins that allow an unimpeded transport of lipids from the prey’s body into the spider gut as part of the solution of digestive fluids and liquefied prey tissues.

#### Proteases – Endopeptidases

Most of the proteases in digestive fluids used for EOD are endopeptidases [24] that break down proteins inside the prey’s body into smaller peptides that can be dissolved and sucked in by the spider. The activity of exopeptidases, on the other hand, is predominantly taking place inside the predator’s gut where the digestion down to amino is finalised. In accordance, expected most of the proteases found in the digestive fluids of our two study were endopeptidases (Tab. 1). The highest abundance among those enzymes was represented by two major clades, serine-proteases (MWROPS: EC 3.4.21) and astacin-like metalloproteases (MWROPS: EC 3.4.24.21). In both our two study species trypsin-like proteases were very abundant, and we could quantify 9 enzymes in *S. mimosarum* and 3 in *A. geniculata*, having concentrations of 6.6% and 8.1% of the digestive fluids, respectively. Three more variants were present in lower concentrations (Tab. 1). Trypsin-like proteases was also one of the major protease groups in digestive fluids of *Nephilingis* [15].

In addition to trypsin-like proteases, we detected astacin-like metalloproteases in large numbers indicating their high importance in EOD. These endopeptidases belong to a large family of zinc-dependent metalloproteases, and several hundreds of these have been identified across the animal kingdom and the bacteria [47, 48, 54]. Apart from digestion, they are also involved in developmental processes and tissue differentiation [55, 56]. Interestingly, especially spider genomes harbour a high number of astacin-like metalloprotease with a highly dynamic evolutionary history of this gene family [10, 15, 57]. Astacin-like metalloproteases were previously shown to be involved in extra-oral digestion [15, 55, 58], a spider key characteristic, and the evolution of this gene family may thus be directly related to the evolutionary success of spiders.

**Fig. 3:**
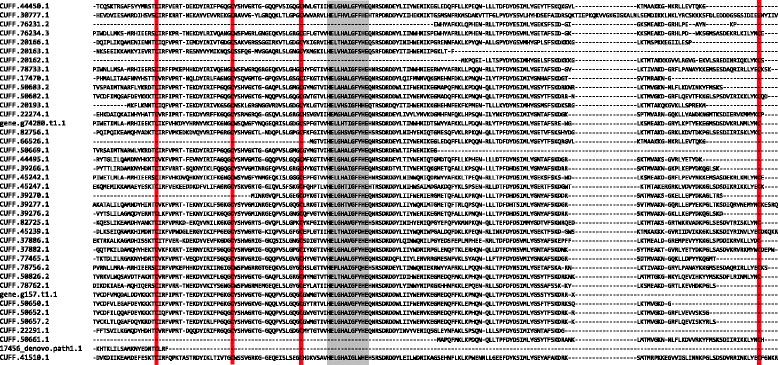
Alignment of protein sequences of all astacin-like metalloproteases found in the genome of *Stegodyphus mimosarum*. Highlighted in *red* are four conserved cysteins, and in *grey* a conserved HEXXHXXGXXHE motif. Both the conserved cysteins and the HEXXHXXGXXHE motif are characteristic for astacin-like metalloproteases (Gomis-Ruth et al. [56])

**Fig. 4:**
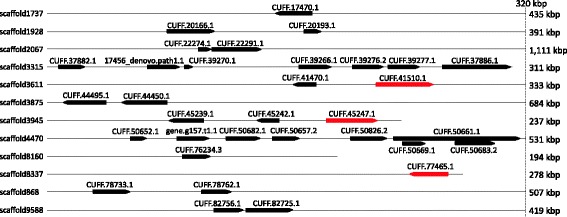
Genomic location of the astacin-like metalloproteases found in *Stegodyphus mimosarum*. Each locus is represented by *black arrows* pointing from 3′ to 5′ end. The total length of each scaffold is written to the right. The entire scaffolds are only shown if they are shorter than 320 kb. Astacins in *red* are present in both, digestive fluid and venom (cf. Additional file 3)

**Fig. 5:**
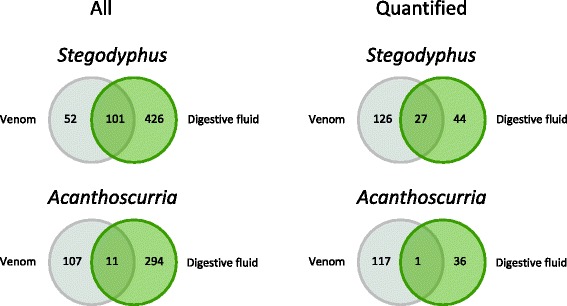
Number of detected proteins in our study species *S. mimosarum* and *A. geniculata* with special respect to overlaps in the compositions of venom and digestive fluid. *Left:* All detected proteins; *Right:* Quantifiable proteins only. There is a significantly greater overlap in *S. mimosarum* (χ^2^ = 89.959, df = 2, *p* < 0.001)

In the genome of *S. mimosarum*, more than 40 astacin-like metalloproteases have recently been identified [10]. Here we show that at least 33 of them are present in the digestive fluid of this species (9 of those were semi-quantified, to a concentration of 7.2% of the digestive fluid). Similarly, the *A. geniculata* genome includes a high number of astacin-like metalloproteases, yet their concentration is much lower in the digestive fluid of this species (0.7%). More than 30 astacin-like metalloprotease transcripts were found, however, some redundancy is expected due to the fragmented nature of the *A. geniculata* genome [10]. All astacin-like metalloprotease sequences found in the *S. mimosarum* genome contain the HEXXHXXGXXHE motif, a zinc binding motif, consistent with metalloprotease activity (Fig. 3) [56]. In addition, four conserved cysteines were present that have previously been shown to form disulphide bonds. Twelve astacin-like metalloprotease transcripts were also identified in the digestive fluids of *A. geniculata*, however only 9 of them showed the HEXXHXXGXXHE motif and/or the conserved cysteines included. The high abundance of astacin-like metalloproteases is also confirmed by the study on digestive fluids of *Nephilngis cruentata*, in which Fuzita et al. [15] detected 26 members of this enzyme family. Looking into more detail, we found that the 33 astacins expressed in the digestive fluids of *S. mimosarum* are located on 12 scaffolds, with two scaffolds containing 7 and 8 astacin-like metalloprotease loci (Fig. 4). This suggests that the astacin-like metalloprotease gene family evolved by sequential duplications. A phylogenetic analysis of astacin-like metalloproteases from different chelicerate species conducted by Fuzita et al. [15] suggests that the high number of spider astacin-like metalloproteases are a result of both an ancient duplication specific to spiders, and more recent lineage specific duplications. Finally, three of these proteases were also found in the venom of the species [10] (Fig. 5; Additional file 3).

The impressive abundance of astacin-like metalloproteases in digestive fluids of three spider species representing three different phylogenetic groups (*A. geniculata* - Mygalomorphae; *S. mimosarum* - Araneomorphae, Eresidae; and *N. cruentata* - Araneomorphae, Araneoidea, cf. Fig. 2 and [15]), suggest that this protein family is essential for successful extra-oral digestion in spiders and therefore widespread. The finding of Fuzita et al. [15] that it is mainly Ast1-type astacin-like metalloproteases (Ast1a, Ast1b and Ast1c) suggests that this type may be responsible for the success of EOD. However, the specific roles or targets of astacin-like metalloproteases in EOD are still unknown. Members of this protein family lyse the egg chorion in seahorses leading to hatching [57]. In the parasitic nematode *Caenorhabditis elegans,* astacin-like metalloproteases break down connective tissue of its host species [58]. It is tempting to speculate that such a barrier breaking function may be very similar in digestive fluids of spiders, breaking down connective tissues of prey.

#### Proteases – Exopeptidases

As the only members of exopeptidases, we detected two metallo-carboxypeptidases in both of our study species (carboxypeptidase B in *S. mimosarum* and A + B in *A. geniculata*). While we have semi-quantitative data for one enzyme for each species, we found five additional transcripts in *A. geniculata* only (Tab. 1). Metallo-carboxypeptidases are also present in the digestive fluids of *Nephilingis cruentata*. Apart from carboxypeptidases A and B, found in our species, Fuzita et al. [15] also detected carboxypeptidase E (again metallo-) and a serine carboxypeptidase in *Nephilingis*.

### Other digestive fluid proteins

#### Leucine-rich proteins

The most abundant group of proteins apart from the enzymes we could identify consisted of leucine-rich proteins. These proteins contain about 20% of leucine, but their specific function is unclear. There is evidence though, that these proteins play an important role in providing a structural framework for protein-protein interactions [59]. We found no less than 14 leucine-rich proteins in *S. mimosarum*, only four in *A. geniculata*, and seven were detected in *Nephilingis* by Fuzita et al. [15].

#### Highly abundant unknown proteins

More than a third of the digestive fluid in *S. mimosarum* consists of three highly similar proteins that show low similarity to other proteins. We found large quantities of this group (~40% of all quantifiable proteins in the digestive fluid) that shows best blast matches with late embryogenesis abundant proteins (LEA-proteins) in *S. mimosarum*. This protein type was not in the other two species in comparison here. In plants, LEA-proteins are suggested to protect other proteins against degradation and undesired conformational changes due to stress, especially desiccation [60, 61]. The abundant proteins in our study that best matches LEA-proteins, may therefore have a function in protecting enzymes of digestive fluids against degradation, as they are exposed to an unpredictable environment outside the spiders’ body. Moreover, since spiders can starve for several weeks [62, 63], with the next feeding event being unforeseeable, the enzymes have to be kept ready for use in digestive fluids in a water conserving manner, which may explain the necessity of protective proteins.

#### Protease inhibitors

We found different protease inhibitors in the digestive fluids of one of our two study species, *A. geniculata* (3 serpins and 1 cysteine protease inhibitor). Mainly belonging to the superfamily of serpins, their precise function remains hard to determine, as some branches of this protein family are either not yet functionally characterised, or not acting as protease inhibitors at all [64, 65]. While the main function of many serpins is indeed the inhibition of proteases, other regulatory functions are also known, such as blood coagulation, protein folding, viral or parasitic pathogenicity, hormone transport and fibrinolysis [64, 66]. The diversity of secondary functions and the degree of uncertainty about the identity of the serpins we found render the significance of their appearance in digestive fluids inconclusive. In their function as serine protease inhibitors, serpins usually form a covalent complex that irreversibly deactivates the protease [65]. This deactivation, at a first glance, seems counterproductive in a cocktail of enzymes which are intended to quickly dissolve the tissues of prey, however, we suggest that the serpins are nevertheless involved in controlling the proteolytic pathway [67]. Because, there are a few serpins that have been found to also reversibly inhibit proteases [66, 68], and those might possess a preserving function for these enzymes. For the potentially long storage time of digestive fluids in between irregular feeding events, they may delay the action of proteases by keeping them in an inactive precursory state. After decoupling the inhibitor unit from the protein complex, the protease could be activated when needed. Finally, the serpins themselves can be activated and deactivated by different mechanisms like conformational changes, cofactor binding, oxidation or polymerisation [65–67] and thus indirectly control the protease activity in digestive fluids. However, as we were unable to detect the activation status of the serpins, at present it is not possible to provide evidence for this hypothesis.

#### Peroxidases, superoxide dismutases and carbonic anhydrases

In *S. mimosarum* we found a quantifiable amount of a peroxidase in the digestive fluid. Fuzita et al. [15] detected even five in *N. cruentata*. Based on their function to reduce peroxides their effect in digestive fluids may be a detoxification of the liquefied prey ingested by the spiders. The co-occurrence with superoxide dismutases and carbonic anhydrases (catalysing the transformation of carbon dioxide and water to bicarbonate and protons, and back) in both species (cf. Tab. 1), however, suggests an interplay of these enzymes to regulate the pH [69, 70]. This may allow adjusting the optimal pH for the digestive enzymes to function in an unpredictable pH inside the preys’ body. If this hypothesis holds true, it raises the question why all three types of enzymes could not at all be detected in the mygalomorph *A. geniculata* (see discussion below).

#### Toxins

Apart from proteins actively involved in digestion, a few toxins were detected also in the digestive fluids of *S. mimosarum* and *A. geniculata* as well as in the *Nephilingis*-species studied by Fuzita et al. [15]. While this could potentially result from contamination during the digestive fluid sampling, it may also result from the very close anatomical proximity of the openings of the venom ducts of the chelicerae to the mouth of the spiders in a resting position, where contact and mixing of both secretions could have happened prior to the digestive fluid sampling. Alternatively, those toxins may not only be produced by the venom glands but also secreted in the midgut and added to the digestive fluid. Fuzita et al. [15] argue along a similar route after having detected transcripts and proteins corresponding to toxins in the midgut of *N. cruentata*.

### Protein-overlap between venom and digestive fluids

An interesting result of our study is the detected overlap in proteins between venom and digestive fluid in our two spider species, which was previously reported for other species [20, 71]. Previous investigations on the venoms of *S. mimosarum* and *A. geniculata* [10] provide an extensive list of proteins that can be compared with those we here found in the digestive fluids of both species. A protein overlap between both secretions may be functional, because 1) many of the effective molecules in venoms rely on the same mechanisms as digestive enzymes, like proteolysis or matrix dissolution, and 2) the injection of venom precedes the injection of digestive fluids and may be considered as a first step of EOD. Following the same argument, Vassilevski et al. [72] suggested that some active venom components may well function to initiate the digestion process. We put particularly emphasis on physically separating the spider chelicerae and the mouth during the digestive fluid sampling to avoid cross-contaminations of both secretions. Thus, our results support this hypothesis.

In *S. mimosarum*, out of the 153 proteins identified in the venom, 101 of them were also present in the digestive fluid. The other way around, out of the 71 most abundant proteins present in the digestive fluids, 27 were also present in the venom, among them a carbohydrase, a carboxypeptidase, a peroxidase, a nuclease, trypsin-like proteases and astacin-like metalloproteases. The qualitative overlap was thereby different among the different protein groups. For example, only two of the 12 trypsin-like proteases (16.7%) were found in both the venom and the digestive fluid of *S. mimosarum*, while 10 of the 15 detected lipases (66.7%) were present in both secretions (Additional file 3; and cf. Additional file 2 for more details). Interestingly, only 11 of the 118 proteins identified in *A. geniculata* venom were also present in the species’ digestive fluid (2.7%), a significantly lower percentage than in *S. mimosarum* (17.4%; χ^2^ = 89.959, df = 2, *p* < 0.001; Fig. 5). While astacin-like metalloproteases were abundant in digestive fluids, there was only little overlap with the venom (only three Ast1 types are also found in *S. mimosarum* venom). However, these proteases were found to play an important role in venom of *Loxosceles* spiders [19]. The reason why the venom of *S. mimosarum* and *A. geniculata* contain only little or no astacin-like metalloproteases, may be related to the different functioning of their venoms. While *Loxosceles* possesses a necrotic venom, *Stegodyphus* and *Acanthoscurria* use a secretion that is mainly neurotoxic [10], and thus is not predominantly relying on proteolytic reactions.

The smaller overlap in protein composition between venom and digestive fluids in *A. geniculata* perhaps results from differences in the ecology compared to *S. mimosarum*. Both species are generalist predators, yet the latter uses a capture web in trees or shrubs, while the former is free hunting on the ground. *S. mimosarum* can thus be expected to catch a larger number of flying insects (M. Majer pers. comm), while *A. geniculata* will more frequently charge running insects like cockroaches and beetles [28]. As to whether flying and running insects generally differ in certain nutrients has yet to be revealed, the attack mode of both species is truly distinct and reflected by their morphology and their life style. While the smaller *S. mimosarum* spiders usually attack in groups and immobilise their prey by injecting venom, the larger mygalomorph *Acanthoscurria* relies on the power of its fangs to restrain the captured prey. Moreover, the social species has to cope with a higher risk of predation while exposed in the capture web and intra-group competition [73, 74] the digestion process may become subject to selection favouring higher speed of the entire digestion process. In *Acanthoscurria*, where single individuals can monopolise prey items and seek shelter for feeding, the speed of EOD may be less important. We thus suggest that differing demands for speed may provide an explanation for why a number digestive enzymes are already transferred into the preys’ body together with the injection of venom in *Stegodyphus* but not in *Acanthoscurria*.

## General discussion

We analysed the protein composition of digestive fluids of a mygalomorph and an araneomorph spider to evaluate how conserved the contents are across a large distance in the phylogenetic tree. We further assessed the overlap with the proteins present in the venom of both species to consider the potential of venom injections to represent a mode of pre-digestion. We found extensive analogies between the two study species *A. geniculata* and *S. mimosarum,* which suggest that the enzymes in digestive fluids are highly conserved and represent an ancient adaptation to extra-oral feeding in spiders. In this regard, our findings are matching the results on a third species, *Nephilingis cruentata*, by Fuzita et al. [15]. The overlap of digestive enzymes in venom and digestive fluids in *S. mimosarum* further indicates that the injection and subsequent activity of venom can be regarded as a first step of the extra-oral digestion process.

### The EOD process

We propose a sequential action of the protein components found in the digestive fluids according to the stages of the EOD process (Fig. 1). After physically attacking the prey, spiders bite and inject their venom, which in some species may have a pre-digestive effect, owing to trypsin-like proteases, lipases and nucleases. After securing the prey, the spiders release digestive fluids into the prey, which contain a cocktail of various hydrolases and support proteins. Chitinases and other immune related proteins may in this phase act as a first immune response that protects the spiders against bacterial and fungal infections. Certain cathepsins may break down the extracellular matrix while lipases, carbohydrases and proteases start liquefying the prey tissues. In a periodic reflux process, the spiders suck in and regurgitate the mixture to maximise the spread and digestion efficiency (cf. Fig. 1). The EOD finds its completion in the subsequent digestion inside the spider gut, with the resorption of the liquefied prey nutrients in the midgut cells (pinocytosis) and the subsequent intracellular nutrient break down. The time for this process to be completed is a crucial factor for spiders. As the enzymes in use are costly to produce, it is also costly to leave a particular prey item behind before the spiders could regain those proteins by ingesting the majority of the digested prey tissues [24].

Finally, our analyses suggest a significant role of astacin-like metalloproteases in EOD, for example for breaking down connective tissue of prey. We also detected a high number of unknown proteins, therefore further investigations are necessary to more comprehensively unravel the function and activity of spider digestive fluids. Moreover, we found LEA-like proteins in *Stegodyphus,* of which their protein protection function has only been described in plants, but LEA-like proteins may also be important for preserving the functionality of spider digestive fluids. The presence and roles of these and other proteins in spider digestive fluid warrants further examination and verification.

## Conclusions

The main protein families involved in extra-oral digestion are rather conserved in the spider species investigated here, which represent the two main lineages in the spider phylogeny. This suggests that the protein composition of spider digestive fluid is highly adapted to EOD. We could match the function of some proteins to particular stages of EOD. Among the proteases, astacin-like metalloproteases are highly abundant in all species, indicating a high importance of this protein family for the digestion process in spiders. However, we also identify species specific protein compositions. For example, LEA-like proteins are highly abundant in *S. mimosarum*, but absent in the two other species. We further find that a large proportion of the protein overlap between digestive fluid and venom in *S. mimosarum*, but only a small proportion in *A. geniculata*. Those differences may be related to differences in the species’ ecology, yet require further investigations. Finally, the detection of immune related proteins in spider digestive fluid suggests an additional protective function against infections.

## Additional files

Additional file 1: Table S1. Comparative list of identified proteins in digestive fluids of *S. mimosarum* and *A. geniculata*, incl. Accession Number and BLAST-description. (XLSX 23 kb)

Additional file 2: Table S2. Quantification data of identified proteins in all *S. mimosarum* and *A. geniculata* digestive fluid samples. (XLSX 151 kb)

Additional file 3: Table S3. Overview of quantities of proteins detected in both, venom and digestive fluid, for both study species, *S. mimosarum* and *A. geniculata*. (XLSX 12 kb)
